# Skeletal Site-Related Variation in Human Trabecular Bone Transcriptome and Signaling

**DOI:** 10.1371/journal.pone.0010692

**Published:** 2010-05-18

**Authors:** Satya S. Varanasi, Ole K. Olstad, Daniel C. Swan, Paul Sanderson, Vigdis T. Gautvik, Sjur Reppe, Roger M. Francis, Kaare M. Gautvik, Harish K. Datta

**Affiliations:** 1 Musculoskeletal Research Group, Institute of Cellular Medicine, Medical School, Newcastle University, Newcastle Upon Tyne, United Kingdom; 2 Department of Clinical Biochemistry, Oslo University Hospital, Ullevål, Norway; 3 Bioinformatics Support Unit, Institute for Cell and Molecular Biosciences, Medical School, Newcastle University, Newcastle Upon Tyne, United Kingdom; 4 Department of Orthopaedic Surgery, Newcastle General Hospital, Newcastle Upon Tyne, United Kingdom; 5 Institute for Ageing and Health, Newcastle University, Newcastle Upon Tyne, United Kingdom; 6 Institute of Basic Medical Sciences, University of Oslo, Oslo, Norway; 7 Department of Clinical Chemistry, Lovisenberg Deacon Hospital, Oslo, Norway; Deutsches Krebsforschungszentrum, Germany

## Abstract

**Background:**

The skeletal site-specific influence of multiple genes on bone morphology is recognised, but the question as to how these influences may be exerted at the molecular and cellular level has not been explored.

**Methodology:**

To address this question, we have compared global gene expression profiles of human trabecular bone from two different skeletal sites that experience vastly different degrees of mechanical loading, namely biopsies from iliac crest and lumbar spinal lamina.

**Principal Findings:**

In the lumbar spine, compared to the iliac crest, the majority of the differentially expressed genes showed significantly increased levels of expression; 3406 transcripts were up- whilst 838 were down-regulated. Interestingly, all gene transcripts that have been recently demonstrated to be markers of osteocyte, as well as osteoblast and osteoclast-related genes, were markedly up-regulated in the spine. The transcriptome data is consistent with osteocyte numbers being almost identical at the two anatomical sites, but suggesting a relatively low osteocyte functional activity in the iliac crest. Similarly, osteoblast and osteoclast expression data suggested similar numbers of the cells, but presented with higher activity in the spine than iliac crest. This analysis has also led to the identification of expression of a number of transcripts, previously known and novel, which to our knowledge have never earlier been associated with bone growth and remodelling.

**Conclusions and Significance:**

This study provides molecular evidence explaining anatomical and micro-architectural site-related changes in bone cell function, which is predominantly attributable to alteration in cell transcriptional activity. A number of novel signaling molecules in critical pathways, which have been hitherto not known to be expressed in bone cells of mature vertebrates, were identified.

## Introduction

Mechanical loading, and the associated mechanical stress, shows immense skeletal site-related variation [1], [2]. A stark example of such profound differences in the extent of the mechanical loading is that of the lumbar spine (LS), where normally far greater loading is experienced than at such skeletal sites as skull or iliac crest (ILC) [3]. There are therefore marked differences in bone density, micoarchitecture and bone composition at different skeletal sites, which reflect evolutionary adaptation of the skeleton [4]–[6]. A focus of recent interest is how environmental and genetic factors influence bone mineral density (BMD). The genotypic influence on skeleton have largely been focused on the phenotype relating to bone density, and only a handful studies have investigated bone geometry or bone quality indirectly by looking at the risk of fracture [7]. Genetic factors influence bone mass and between 50–90% of variation in BMD is inherited [7]. These BMD determinants are known to be polygenic, and objective evidence for this is provided by candidate gene SNPs (single nucleotide polymorphisms), quantitative trait locus and family linkage studies [7]–[20]. Previous studies have identified multiple candidate genes and chromosomal regions which influence bone mass and are linked to osteoporosis-related phenotypes [10]–[20]. These studies have also provided evidence for the presence of gender- and skeletal site-specific regulation and variation of BMD [10]–[20]. However, the nature of polygenic influences and the resulting complexity of molecular interaction mediating these effects in the development, attainment and subsequent maintenance of bone mineral density (BMD) are unclear.

Bone is a heterogeneous tissue and its histological classification, based on macroscopic texture of cross sections, classifies the tissue into compact (or cortical) and cancellous (or trabecular) subtypes. The trabeculae, within cancellous bone, while may appear to be disorganized are in fact arranged to provide maximum strength and are aligned in the direction to support maximum mechanical stress. Trabecular bone has a porous network of irregular cavities, and being less dense than compact bone, constitutes only a small fraction of total bone mass, but provide approximately 90% of the total bone surface area representing about 80% of the exchangeable calcium pool. It is this much larger surface area that makes trabecular bone metabolically more active than compact bone. The question as to how mechanical loading, and the associated mechanical stress, affects particular histological type of bone at different sites in the skeleton is of interest. To date many studies have been performed examining the mechanical stress related changes in the bone strength, microarchitecture and even cellular changes [1]–[6]. The effect of differences in the mechanical loading on the underlying complex molecular architecture of bone, which is critical for bone function, has never been investigated. Therefore, to address this question we have performed a global gene transcriptome investigation on human male cancellous bone from two anatomical sites, exposed to marked differences in mechanical and functional demands.

## Results

### Raw data Analysis

In view of high degree of molecular homogeneity in the overall transcript expression between these sites (>95%), the data from the three lumbar vertebrae, namely L2, L3 and L4, were grouped for analysis (Table S1). If filtration on absents (or presents) is used to remove transcripts that have absent call in all the arrays then 40,450 transcripts were found, and the number of transcripts expressed in all of the bone biopsies is approximately 14,300. The Venn analysis revealed 9177 gene transcripts to be uniquely expressed in the LS as opposed to 713 gene transcripts in the ILC. A statistical comparison of global gene expression in lamina of lumbar vertebrae (L2, L3 and L4) and ILC revealed differential expression of 4244 gene transcripts with significant differences (fold change ≥2.0, p-value ≤0.05). It was seen that compared with ILC, in LS region 3406 transcripts were up-regulated and 838 were down regulated. However, the number of differentially expressed transcripts between the skeletal sites was found to be 1733 for an adjusted p-value <0.05 and fold change >2; 1025 probesets are up-regulated in LS versus ILC, 708 probesets are downregulated in LS compared to ILC. Examination of Gene Ontology (GO) terms show a statistically significant over representation for a number of GO terms (p<0.005) including biological and cell adhesion, extracellular matrix formation and skeletal development (Table S2).

### Bone cells related transcriptomes

The detailed analysis of the data reveals some profound and consistent changes in the transcriptomes that are characteristically associated with skeletal tissues, which were up-regulated in the LS when compared with ILC (Table 1 & 2). Bone related genes, such as SOST, MEPE and matrix Gla protein were among the most up-regulated, but also novel and genes with uncharacterised gene function, such as Zic1, cDNA clone ZA88B06 and DKFZP434C153 protein showed high differential expression. The comparison of transcripts characteristically associated with osteoblasts, osteoclast and osteocytes, revealed profound difference between the ILC and LS (Table 3). Interestingly, whilst transcripts of enzymes, matrix proteins and humoral factors, reflecting activities of osteoblasts, osteoclasts and osteocytes showed up-regulation in the LS, the cell structure components of these cells, namely PTHR2, CTR and PDPN, respectively, showed similar expression in both sites. The array data was validated by qRT-PCR of RNA from five ILC and equal number of randomly selected LS biopsies. The verification was carried out by determining expression of select group of differential expressed transcripts. The transcripts were selected to reflect respective expression activities of each of the three bone cell types, for osteoclast CATK, for osteoblast Col1α and for osteocytes SOST, DMP1 and MEPE (Table 3 & 4). The increased expression in the LS compared with ILC suggested increased transcriptional activity of the three bone cells rather than any changes in their numbers. OSCAR and PTHR2 expression seen in the array data were substantiated by qRT-PCR, which reflects lack of site-related significant differences in actual number of osteoclast and osteoblast respectively. Thus, the major molecular differences between the two distinct bone sites were strictly associated with osteocyte and osteoblast related transcripts encoding matrix (secretory) proteins and key master regulatory genes encoding transcription factors, e.g. RUNX2.

**Table 1:** Top up-regulated genes showing up-regulation >50 fold in lamina of lumbar vertebrae (LS) in comparison with iliac crest (ILC).

Probe Set ID	Gene Title	Gene Symbol	p-value LS vs. ILC	FC Absolute LS vs. ILC	S.D
206373_at	zic1 family member (odd-paired homolog, Drosophia)	Zic1	8.95E-08	202.03	1.78
217404_s_at	collagen, type II, alpha 1 (primary osteoarthritis, spondyloepiphyseal dysplasia, congenital)	COL2A1	1.6E-5	167.51	2.95
205713_s_at	cartilage oligomeric matrix protein	COMP	1.08E-11	166.98	1.15
209904_at	troponin C type 1 (slow)	TNNC1	0.003	157.67	4.79
206227_at	cartilage intermediate layer protein, nucleotide pyrophosphohydrolase	CILP	6.84E-11	154.45	1.25
214087_s_at	myosin binding protein C, slow type	MYBPC1	1.87E-04	134.89	3.41
204737_s_at	myosin, heavy chain 6, cardiac muscle, alpha (cardiomyopathy, hypertrophic 1) myosin, heavy chain 7, cardiac muscle, beta	MYH6///MYH7	0.002	112.49	4.18
204810_s_at	creatine kinase, muscle	CKM	0.002	96.48	4.07
209888_s_at	myosin, light chain 1, alkali; skeletal, fast	MYL1	8.70E-04	84.64	3.58
209742_s_at	myosin, light chain 2, regulatory, cardiac, slow	MYL2	0.002	82.85	3.88
213201_s_at	troponin T type 1 (skeletal, slow)	TNNT1	0.002	79.48	3.99
209621_s_at	PDZ and LIM domain 3	PDLIM3	3.82E-05	77.27	2.68
213782_s_at	myozenin 2	MYOZ2	0.002	74.63	3.89
212654_at	tropomyosin 2 (beta)	TPM2	3.23E-04	69.76	3.14
213492_at	collagen, type II, alpha 1 (primary osteoarthritis, spondyloepiphyseal dysplasia, congenital)	COL2A1	4.40E-05	68.00	2.49
222043_at	Clusterin	CLU	1.32E-09	62.26	1.31
203872_at	actin, alpha 1, skeletal muscle	ACTA1	0.00307	60.50	3.85
219728_at	Myotilin	MYOT	8.21E-04	58.01	3.31
205054_at	Nebulin	NEB	0.001	57.24	3.31
204179_at	Myoglobin	MB	0.001	56.32	3.51
228224_at	proline/arginine-rich end leucine-rich repeat protein	PRELP	2.74E-09	55.65	1.19
219106_s_at	kelch repeat and BTB (POZ) domain containing 10	KBTBD10	0.002	50.02	3.41

FC, fold change; SD, standard deviation.

**Table 2:** Selected skeletal genes showing significant up-regulation on expression in the lamina of lumbar vertebrae (LS) compared with iliac crest (ILC).

Probe Set ID	Gene Title	Gene Symbol	*p-*value LS *vs. ILC*	FC Absolute LS *vs. ILC*	S.D.
223869_at	Sclerosteosis	SOST	1.90E-08	40.79	1.35
208175_s_at	dentin matrix acidic phosphoprotein	DMP1	1.35E-05	13.59	1.37
221150_at	matrix, extracellular phosphoglycoprotein with ASARM motif (bone)	MEPE	2.60E-05	9.22	1.30
231766_s_at	collagen, type XII, alpha 1	COL12A1*	2.51E-04	12.77	1.82
231879_at	collagen, type XII, alpha 1	COL12A1*	0.00182	4.141	1.23
225664_at	collagen, type XII, alpha 1	COL12A1*	2.39E-07	13.26	1.05
209101_at	connective tissue growth factor	CTGF	2.41E-07	11.77	1.05
238481_at	matrix Gla protein	MGP*	8.48E-07	20.55	1.24
202291_s_at	matrix Gla protein	MGP*	7.42E-09	12.45	0.85
232267_at	G protein-coupled receptor 133	GPR133	2.30E-10	14.34	0.72
219522_at	four jointed box 1 (Drosophila)	FJX1	8.63E-06	7.75	1.02
1561754_at	Full length insert cDNA clone ZA88B06	ZA88B06	1.41E-04	4.05	0.93
241902_at	mohawk homeobox	MKX*	2.55E-04	7.82	1.46
239468_at	mohawk homeobox	MKX*	5.28E-06	6.55	0.88
213591_at	aldehyde dehydrogenase 7 family, member A1	ALDH7A1	5.40E-05	8.08	1.24
208950_s_at	aldehyde dehydrogenase 7 family, member A1	ALDH7A1	5.76E-04	2.22	0.60

FC, fold change; SD, standard deviation.

*Multiple probe sets.

**Table 3:** Comparison of differences in the absolute expression of osteoblast, osteoclast and osteocyte-related gene transcripts in lamina of lumbar vertebrae (LS) and iliac crest (ILC).

Cell type	Probe Set ID	Gene Symbol	Gene Title	FC (LS vs. ILC)
Osteocytes	208175_s_at	DMP1	dentin matrix acidic phosphoprotein	13.59
	221150_at	MEPE	matrix, extracellular phosphoglycoprotein with ASARM motif (bone)	9.22
	221166_at	FGF23	fibroblast growth factor 23	3.52
	223869_at	SOST	sclerosteosis	40.79
	204879_at	PDPN*	podoplanin	6.43
	208233_at	PDPN*	podoplanin	2.82
	221898_at	PDPN*	podoplanin	5.90
	226658_at	PDPN*	podoplanin	2.75
Osteoblasts	202310_s_at	COL1A1*	collagen, type I, alpha 1	6.13
	202311_s_at	COL1A1*	collagen, type I, alpha 1	3.99
	1556499_s_at	COL1A1*	collagen, type I, alpha 1	2.19
	206956_at	BGLAP	bone gamma-carboxyglutamate (gla) protein (osteocalcin)	3.78
	209875_s_at	SPP1	secreted phosphoprotein 1 (osteopontin, bone sialoprotein I, early T-lymphocyte activation 1)	2.97
	212667_at	SPARC*	secreted protein, acidic, cysteine-rich (osteonectin)	5.86
	200665_s_at	SPARC*	secreted protein, acidic, cysteine-rich (osteonectin)	4.01
	209875_s_at	SPP1	secreted phosphoprotein 1 (osteopontin, bone sialoprotein I, early T-lymphocyte activation 1)	2.97
	207370_at	IBSP*	integrin-binding sialoprotein (bone sialoprotein, bone sialoprotein II)	3.97
	236028_at	IBSP*	Integrin-binding sialoprotein (bone sialoprotein, bone sialoprotein II)	7.35
	236859_at	RUNX2*	runt-related transcription factor 2	3.80
	221283_at	RUNX2*	runt-related transcription factor 2	2.74
	236858_s_at	RUNX2*	runt-related transcription factor 2	2.21
	205289_at	BMP2*	bone morphogenetic protein 2	2.71
	205290_s_at	BMP2*	bone morphogenetic protein 2	2.60
	239769_at	CDH11*	Cadherin 11, type 2, OB-cadherin (osteoblast)	3.00
	207173_x_at	CDH11*	cadherin 11, type 2, OB-cadherin (osteoblast)	2.85
	205911_at	PTHR1	parathyroid hormone receptor 1	4.29
	206772_at	PTHR2	parathyroid hormone receptor 2	−1.59
Osteoclasts	202450_s_at	CTSK	cathepsin K	4.04
	1554503_a_at	OSCAR	osteoclast associated, immunoglobulin-like receptor	−1.28
	207886_s_at	CALCR*	calcitonin receptor	1.59
	207887_s_at	CALCR*	calcitonin receptor	1.04
	204638_at	ACP5	acid phosphatase 5, tartrate resistant	2.03

FC, fold change.

*Multiple probe sets.

**Table 4:** The validation of the array data was carried out by qRT-PCR of select number of transcripts for equal number of iliac crest (ILC) and lumbar spine (LS) samples (n = 5).

Gene	Affymetrix Absolute Fold Change (ILC vs. LS)	qRT-PCR RQ (ILC vs. LS)
COL1A1	−7.2	−22.8
SOST	−53	−90.7
DMP1	−42	−33.0
MEPE	−23.5	−40.7
CTSK	−8.2	−15.8
ZIC1	−86.7	−666
PTHR2	1.5	1.7
OSCAR	1.7	1.4

### Pathways analysis and novel gene identification

**Figure 1:**
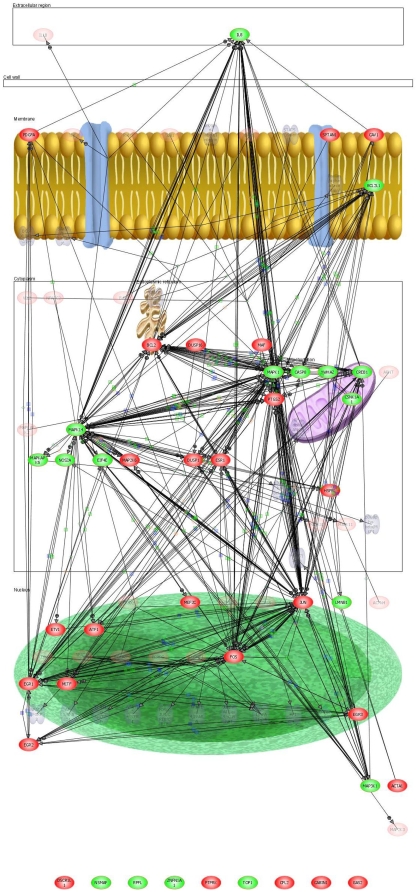
TNF receptor signaling in trabecular bone. Direct interaction network models were constructed by Pathway Architect analysis of the differentially expressed transcript in the lumbar spine and iliac crest trabecular bone. Up-regulated transcripts are indicated red whilst the down-regulated are shown in green. Arrows link interacting genes and positive (+) and negative (−) associations are marked respectively. Green boxes denote regulation, blue boxes binding and orange circles indicating phosphorylation.

**Figure 2:**
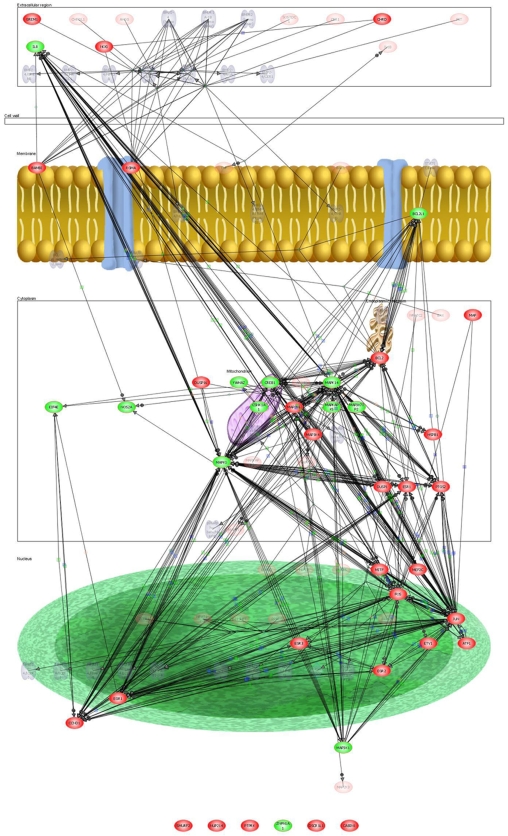
BMP signaling in trabecular bone. In this interaction network model, constructed by Pathway Architect analysis software of differential expressed genes between iliac crest and the lumbar spine, up-regulated transcripts are indicated red whilst the down-regulated are shown in green. Arrows link interacting genes and positive (+) and negative (−) associations are marked respectively. Green boxes denote regulation, blue boxes binding and orange circles indicating phosphorylation.

**Figure 3:**
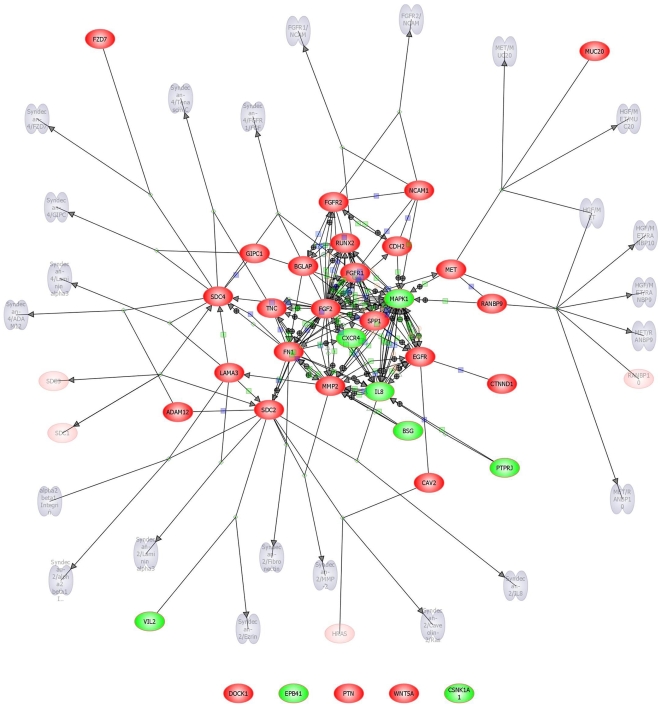
Proteogylcan Syndecan-mediated signaling in trabecular bone. Direct interaction network models constructed by Pathway Architect analysis of the differentially expressed transcript in the lumbar spine and iliac crest trabecular bone. Up-regulated transcripts are indicated red whilst the down-regulated are shown in green. Arrows link interacting genes and positive (+) and negative (−) associations are marked respectively. Green boxes denote regulation, blue boxes binding and orange circles indicating phosphorylation.

The differential analysis revealed that the top three pathways, i.e., with the highest number of differentially expressed transcripts, were TNF receptor signaling pathway, BMP signaling and Proteogylcan syndecan-mediated signaling (Tables S3 to S6; Figures 1, 2, & 3). The details of the gene transcripts for the signaling mediated by TNF receptor, BMP and Proteogylcan syndecan-mediated pathway (Figures 1, 2 & 3) is given in Tables S4, S5 and S6, respectively. The majority of the post-receptor transcripts of these pathways were found to show up-regulation in the LS when compared with ILC. The analysis also reveals a number of hitherto unidentified and unknown transcripts which are involved in the signaling.

**Figure 4:**
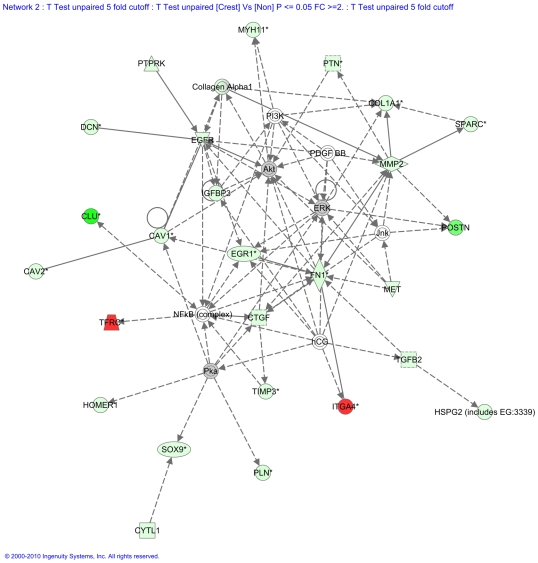
Network enriched for genes involved in skeletal system. Molecules are represented as nodes, and the biological relationship between two nodes is represented as an edge (line). The intensity of the node color indicates the degree of down- (red) or up- (green) regulation where lumbar spine is compared with iliac crest. Nodes are displayed using various shapes that represent the functional class of the gene product. Direct relationships are shown in solid arrows, indirect relationships in dashed arrows. Genes with no colour are added to the network by Ingenuity Pathway Analysis as part of the network generation algorithm.

Probesets with a 5-fold or more change in expression were analysed with Ingenuity Pathway Analysis (IPA), a list of 268 eligible entities for IPA analysis. The most significant biological function classification was in skeletal and muscular development and function with the most significant subclass being skeletal development (corrected p-value of 2.22×10^−9^) (Table S7). Network analysis of the data set suggested one interaction network with skeletal and muscular system development and function including the genes Akt, COL1A1, CTGF, EGFR, EGR1, ERK, IGFBP3, ITGA4, Jnk, MYH11, NF-kB, (complex), PDGFBB, PI3K, Pka, POSTN, PTPRK, SOX9, SPARC, TGFB2, TIMP3 (Figure 4).

## Discussion

The global transcriptosome first ever analysis of human lamellar LS and ILC revealed that whilst most of the transcripts showed a similar level of expression, a limited number of functionally associated subsets showed marked anatomical site-related differences. Interestingly, between L2, L3 and L4 vertebrae from different subjects there was a higher overall molecular homogeneity when compared with ILC from the same person. Therefore for further comparison the three vertebrae were treated as one group. Similarly, high degree of overall molecular homogeneity in gene expression was seen between symmetrical sites in ILC [20] underscoring the importance of function as a major determinant of bone cell gene expression .and adaptability. The number of gene transcripts and the extent of their up-regulation was much higher in the lamina of spinal vertebra when compared with ILC. The analysis of up-regulated genes showed that these were related to changes in key cellular and molecular components and strongly associated with essential biological functions in bone. The gene transcripts, reflecting respective transcriptional activities of osteocytes, osteoblasts and osteoclasts from vertebrae when compared with ILC, were found to be consistently up-regulated in the vertebrae and magnitude of the increased expression was quite similar. In contrast, the gene transcripts that reflect osteocytes, osteoblasts and osteoclasts number showed insignificant or less marked skeletal site-related variation. This conclusion was based on an analysis of the data evaluating osteocyte-, osteoblast- and osteoclast-specific structural transcripts, namely podoplanin in osteocytes, parathyroid hormone receptor in osteoblasts and osteocytes and calcitonin receptor and osteoclast-associated receptor in osteoclasts. We postulate that these transcripts provide estimate of the cell numbers with the reasonable assumption that each cell type in vertebra and ILC express on average similar number of the marker molecules. Since these transcripts were found to show only minor differences in vertebra and ILC when compared with other activity related transcripts (Table 3 & 4), we interpret that the observed increase in the respective activities of the three bone cell types were predominantly due to increase in the relative cell activity rather than reflecting a change in absolute cell number. Interestingly, the most profound up-regulation in the spinal vertebrae was observed for transcripts reflecting osteocyte activity, namely in SOST, DMP1, and MEPE.

In light of the recent observations, the data analysis allows us to suggest possible mechanisms for the existing evidence of increased bone turnover, based on the described molecular differences in osteoblast- and osteoclast-related bone remodelling in lamellar bone of LS when compared with ILC. It is generally accepted that mechanical stress induced by weight-bearing exercise increases osteoblast activity, and that the absence of mechanical stimulation resulting from prolonged immobilization or microgravity causes severe bone loss [21], [22]. In recent years a number of observations suggest, based on *in vitro* studies and animal transgenic models, that osteocytes act as mechano-sensory cells and that lacuno-canaliculi carry signaling molecules that are responsible for maintenance of bone structure and mass [23]. Osteocytes are interconnected by a network that involves their dendritic processes within lacunae to osteoblasts; that mirrors the CNS neuronal network, but comprehensive identity of all relevant signaling molecules in bone remains to be identified [24], [25]. We believe that the present data show that osteocytes are more metabolically active than hitherto understood and play a central role in the determination and maintenance of bone structure [24]–[26]. The bone matrix secludes and isolates osteocytes which have an extensive interaction and also connect to other surrounding cells via an elaborate network of dendritic processes which may have the potential to modulate bone resorption [26].

**Figure 5:**
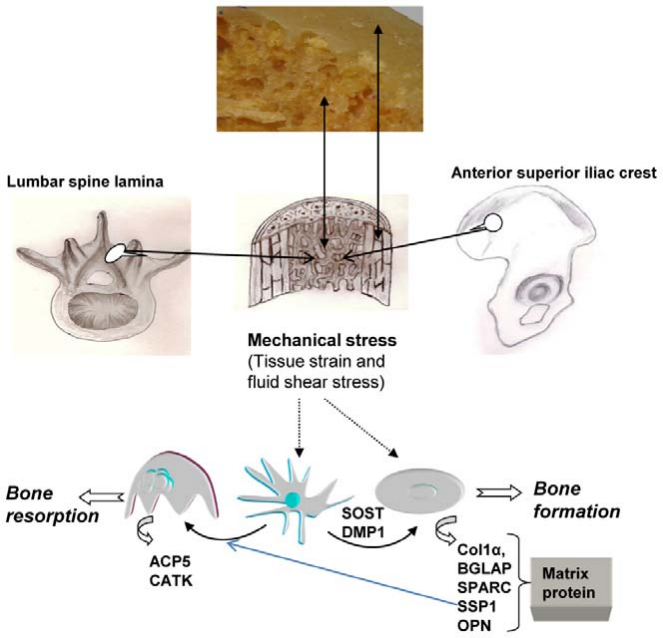
Role of osteocytes in mechanotransduction in trabecular bone. The increased mechanical stress is detected by mechanosensors, a function which is primarily performed by the osteocytes (∼90% of the total cell number). The increased mechanical stress is mechano-transduced into intracellular biochemical signals by osteocytes and results in increased transcriptional activity of range of genes (SOST, MEPE and DMP1). Osteocytes also transmute mechanical stress into intercellular biochemical signals to modulate the respective activities of the osteoclasts and osteoblasts.

We hypothesize that significantly higher transcriptional activity within specific functional osteocyte networks in the spinal vertebra, when compared with ILC, is reflective of the increased stress experienced at the LS [3]. The mechanical stress, both due fluid shear stress and tissue strain, in bone is now thought to be mainly detected by osteocytes (Figure 5) [24]–[29]. Osteocytes are involved in mechano-sensing as well as in mechano-transduction of the stress into biochemical signal [24]–[29]. The increased expression of osteocyte-related transcripts, such as SOST, DMP1, MEPE, in the spine relative to the ILC, provides for the first time evidence of mechanical stress-related *in vivo* increase in osteocyte transcriptional activity. The osteocyte activity, resulting from excess mechanical loading, increases the respective activities of osteoblasts and osteoclasts, as evident from increase in the expression of matrix proteins (COL1A, SPARC, and IBSP) and osteoclast-specific enzymes (CTSK and ACP5) (Table 3 & 4; Figure 5). These genes, i.e., characteristically associated with osteocyte, osteoblasts and osteoclasts, showing markedly increased expression, encode secretory proteins. Further evidence of skeletal site-related difference in the bone remodelling activity due to mechanical stress, that seem to be orchestrated by osteocyte, is provided by the PathwayArchitect™ analysis (Figures 2, 3 and 4; Tables S4, S5 and S6). The analysis shows that top pathways, in terms of differential expression in the lamina of LS as compared with the ILC, are those involved in the bone formation by osteoblasts (BMP signalling pathway, Proteoglycan Syndecan signalling) and bone-resorption by osteoclast (TNF receptor signalling, and other network groups, e.g. adherence/adhesion). The most likely explanation in the observed increase in transcriptional activity in the osteocyte, as well as osteoblasts and osteoclasts, in the spine is likely to be a higher degree of local mechanical stress [3]. The data presented provides support for the proposal that osteocytes modulate respective bone-formation and bone-resorptive activities of osteoblasts and osteoclasts, and thereby regulate and maintain of bone mass. These observations add to our understanding about the central role of osteocytes in the maintenance of bone mass in health and in the pathogenesis of osteoporosis.

The comparison of the relative expression of the transcripts led to identification of a number of novel and uncharacterised genes, whose function in bone cell biology is not yet known [30], [31]. In addition, the pathways analysis revealed the known key signaling molecules (Figs. 1, 2 & 3 and corresponding Tables S4, S5 & S6), and led to the identification some novel pathways specific intracellular putative proteins. Further studies, such as cell protein expression, protein-protein interaction and knock-in and knock-out functional studies are required to establish the role of these novel proteins. In conclusion, taken together the data show that at the transcriptional level bone has a strong ability to adapt to functional demands, and osteocyte play a critical role in the process. The adaptive response involves “growth and developmental processes”, probably made possible through their “high sensitivity to synaptic processes” and “matrix” engineering properties.

## Methods

### Subject selection

The participants numbered thirteen Caucasian men who were all from North East England, U.K., who were undergoing spinal decompression laminectomies or spinal fusions of the LS. This study was conducted according to the principles expressed in the Declaration of Helsinki. The study was approved by the Institutional Review Board of Northumberland Local Research Ethics Committee, Blyth, Northumberland NE24 2AG (REC Reference: 04/Q0902/29), and all subjects gave their informed written consent. Underlying secondary causes of osteoporosis were excluded by medical history, physical examination and laboratory investigations. Men with a history of treatment with antiresoptive agents, steroid, anticonvulsant and anticoagulant were excluded [32] (Text S1). The mean±SD (range) of age, weight and height respectively were 53.9±10.7 (35.0-69.0)yr, 86.6±8.6 (84.0-108.2)kg and 176.4±6.3 (167.0-184)cm, respectively. The mean±SD (range) for BMD, given as areal density, for LS, total hip, and femoral neck respectively were 1.067±0.332 (0.760-1.387), 1.047±0.153 (0.820-1.271) and 0.870±0.153 (0.620-1.202) gm/cm^2^, respectively. None of the subjects had history of low trauma fractures (Table S8 and Text S1).

All bone biopsies were trabecular and taken from a specific site in the ILC site, 2 cm backwards from the anterior superior iliac spine. Further consistency was achieved by ensuring that only one senior surgeon supervised all biopsies with explicit instructions to avoid attached tissues, specifically muscle. Similarly spinal biopsies were all trabecular and all were from lamina processes of the lumbar vertebrae two, three and four. A total of 24 biopsies were obtained for analysis from 13 men, 19 from the lamina of the LS and 5 from the ILC.

### Biochemical and BMD measurements

Serum testosterone, sex-hormone-binding globulins, follicular stimulating hormone and luteinizing hormone were measured by commercially available radioimmunoassays (SAS laboratory, Royal Victoria Infirmary, Newcastle upon Tyne). All bone density measurements were performed by DXA using a Hologic QDR 2000 Bone Densitometer (Hologic, Waltham, MA). In vivo precision for measurement with this system is 1.0% at the LS (L1-L4) and 1.5% for the femoral neck. BMD results were obtained as an areal density in g/cm^2^, but were also given as T- and Z- scores. The T-score is the number of standard deviation units above or below the mean for normal young men, whilst the Z-score is the number of standard deviation units above or below the age-related normal men (calculated using the manufacturer's standard normal reference database). The results of laboratory investigations for all these subjects were within normal laboratory ranges.

### Extraction of RNA

The trabecular bone from the laminar and ILC that were excess to the surgical requirement were collected as biopsies of size approximately 0.25 to 1 cm^3^. The biopsies were immediately frozen and stored in liquid nitrogen for later extraction of RNA. The frozen bone biopsies were pulverized with a mortar in liquid nitrogen with their content of marrow intact. RNA was then extracted by homogenization in Trizol (Life Technologies, Invitrogen, cat no 15596) 1 ml/100 mg, then following the manufacturers procedure. RNA was further purified using the RNeasy kit (Qiagen) to remove organic components and finally re-suspended in RNAse free deionsed water and quantified using the Nanodrop spectrophotometer. The total RNA integrity was checked using Agilent 2100 BioAnalyzer (Agilent Technologies, Inc.) prior to cDNA synthesis. In addition, the quality of the RNA was controlled according to the Affymetrix test manual by measuring the ratio between 3′ and 5′ end for GAPDH mRNA (ratio always<2.0)

### Microarray analysis

Double-stranded cDNA and biotin-labeled cRNA probes were made from 5 µg total RNA using the Superscript Choice system (Invitrogen) and the Enzo Bioarray respectively. Procedures were performed according to recommendations from Affymetrix [20], [31]. This cRNA was hybridized to Affymetrix Human Genome U133 Plus 2.0 Array containing cDNA oligonucleotides representing more than 54,000 probe sets for 38,000 different genes followed by washing and staining on the GeneChips Fluidics Station 450 (Affymetrix) according to manufacturer's instructions. The chips were scanned on the Affymetrix GeneChip® 3000 scanner. The quality of the RNA and probe was controlled by an Affymetrix based test measuring the ratio between 5′ and 3′ mRNAs for β-actin and GAPDH and found to be highly satisfactory. The datasets originating from the specimens were first processed by the Affymetrix Mas5.0 software, and signal values representing the expression level of each transcript were generated. Each sample was normalized as recommended by Affymetrix by multiplying all signal values with a scaling factor that results in an average signal value of 500 for all genes that are classified as “present” calculated by the Affymetrix MAS 5.0 program. The scaling factor is set by examining all the probe sets on the array to compute a trimmed mean signal and derive a scale factor for the array so that: Target signal  =  Scale factor × Trimmed mean signalprobe array. Thus, the scale factor standardizes the trimmed mean signal of the array to the target signal. The patients were coded, and all analyses were carried out blindly. Both samples from each patient were analyzed in the same kit for cRNA probe synthesis and hybridized to chips from the same batch. One chip was used for each sample (cRNA synthesis) as the variability between chips and cRNA syntheses is significantly lower than the potential variability derived from different biological samples.

### Data Analysis

The overview of differentially expressed genes in the LS relative to ILC in male controls was generated by the use of Affymetrix software, which made it possible to compare data from two arrays. The statistical analysis is based on 22 different cDNA oligonucleotides to measure quantitatively one mRNA transcript, and each cDNA probe is distributed as 22 different “micro-spots”. Thus, 22 signals for each mRNA transcript (probeset) are generated and enables the Affymetrix software GCOS to compute p-values for differential expression when two transcripts on two different arrays are compared. The Wilcoxon's Signed Rank test uses the differences between Perfect Match and Mismatch probe signal intensities, as well as the differences between Perfect Match intensities and background to compute each p-value difference. From Wilcoxon's Signed Rank test, a total of three, one-sided p-values are computed for each probe set. The most conservative value is chosen to determine the “change call”. That is the value closest to 0.5 signifying that no change is detected. These are combined to give one final p-value.

Data from HGU-1333 plus 2 Affymetrix GeneChip arrays was imported into GeneSpring GX 11 and summarised and normalised with MAS5 and GC-RMA. Flag data from the MAS5 analysis was used to derive a probeset list where at least 19 out of 25 samples had a present or marginal call. This left 20796 probesets, which was further reduced to 20764 probesets after Affymetrix control probesets were removed from the list. This list was used for all downstream statistical analysis. An unpaired t-test between LS and ILC samples was used to detect differential expression. Probesets reported as being differentially expressed if they satisfy a corrected p-value cutoff of <0.05 when performing multiple testing correction with Benjamini-Hochberg False Discovery Rate (FDR).

Pathway analysis was also carried out with Ingenuity Pathway Analysis 8.5 (Ingenuity Systems http://www.ingenuity.com) on probesets differentially expressed in lumbar and ILC biopsies. Canonical pathways analysis identified the pathways from the Ingenuity library of pathways that were most significant to the data set. The significance of the association between the data set and the canonical pathway was measured in 2 ways: 1) A ratio of the number of molecules from the data set that map to the pathway divided by the total number of molecules that map to the canonical pathway is displayed. 2) Fisher's exact test was used to calculate a p-value determining the probability that the association between the genes in the dataset and the canonical pathway is explained by chance alone.

Affymetrix identifiers and fold change information was loaded into IPA and each identifier was mapped to its corresponding object in Ingenuity's Knowledge Base. A 5-fold cutoff of fold change was set to identify molecules whose expression was significantly differentially regulated. These molecules, called Network Eligible molecules, were overlaid onto a global molecular network developed from information contained in Ingenuity's Knowledge Base. Networks of Network Eligible Molecules were then algorithmically generated based on their connectivity. The Functional Analysis of a network identified the biological functions and/or diseases that were most significant to the molecules in the network. The network molecules associated with biological functions and/or diseases in Ingenuity's Knowledge Base were considered for the analysis. Right-tailed Fisher's exact test was used to calculate a p-value determining the probability that each biological function and/or disease assigned to that network is due to chance alone. All edges of the pathways generated are supported by at least one reference from the literature, from a textbook, or from canonical information stored in the Ingenuity Pathways Knowledge Base. Human, mouse, and rat orthologs of a gene are stored as separate objects in the Ingenuity Pathways Knowledge Base, but are represented as a single node in the network.

ArrayAssist (Stratagene) was further used to created a raw data set and identify differential gene expression. The change in relative expression of the remaining set of transcripts was assessed using t-tests with Benjamin-Hochberg (false discovery rate, FDR) correction (2-fold minimum cut-off and p<0.05). These transcripts, i.e., showing FC≥2 in bone biopsies taken from ILC and LS, were further analysed by PathwayArchitect™ software (Stratagene). This analysis utilizes existing relevant literature for pathway analysis and visualization and permits identification of shared common direct regulators or downstream targets. PathwayArchitect™ analysis software can identify gene-gene interaction, gene regulation and their key functions in the complex pathways.

The guidelines described in MIAME (Minimal Information About a Microarray Experiment) has been followed in writing this paper. The primary data has been submitted to the European Bioinformatics Institute (EMBL-EBI) ArrayExpress repository. The experiment name is “Skeletal Site-Related Variation in Human Bone Transcriptome” and Signalling ArrayExpress accession number is: E-MEXP-2219.

### Quantitative RT-PCR

The Affymetrix gene expression data were validated for selected transcripts using the TaqMan gene expression assays and the Applied Biosystems Prism 7900 HT sequence detection system. Five 500 ng total RNA from each donor was reverse transcribed using Omniscript (Qiagen Ltd.), and cDNA representing 2.5 ng total RNA was used in each PCR reaction. The PCR reactions were run in duplicates. The relative changes of each transcript, using GAPDH (glyceraldehyde-3-phosphate dehydrogenase) as endogenous control, were calculated using the 2(ΔΔC(T) method [33], and the gene expression results are given as RQ (relative quantitation).

## Supporting Information

Table S1 Anatomical-site related comparison of overall molecular homogeneity between different skeletal sites.
(0.01 MB .DOCX)

Table S2 Examination of Gene Ontology (GO) terms shows an overrepresentation for a number of GO terms (p<0.005) including cell adhesion, extracellular matrix formation and skeletal development.
(0.03 MB XLS)

Table S3 Top 30 signalling pathways identified by comparing differential transcript expression in the lumbar spine versus iliac crest (4244 gene transcripts with FC ≥2, p-value ≤0.05).
(0.06 MB DOC)

Table S4 TNF receptor signalling pathway gene transcripts identification based on differential expression in lamina lumbar spine and iliac crest; the analysis was carried out using Pathway Architect Software and the pathway generated is shown in Fig. 2.
(0.06 MB DOC)

Table S5 BMP signalling pathway gene transcripts identification based on the differential expression in iliac crest and in the lamina of lumbar spine; the analysis was carried out using Pathway Architect Software and the pathway generated is shown in Fig. 3.
(0.06 MB DOC)

Table S6 Proteoglycan Syndecan signalling events identification based on analysis of differential expression in the lamina of lumbar spine and iliac crest; the analysis was carried out using Pathway Architect Software and the pathway generated is shown in Fig. 4.
(0.06 MB DOC)

Table S7 Probesets with a 5-fold or more change in expression were analysed with Ingenuity Pathway Analysis (IPA), a list of 268 eligible entities for IPA analysis. The most significant biological function classification was in skeletal and muscular development and function.
(0.04 MB XLS)

Table S8 Anthropometric indices age and bone density of individual subjects.
(0.05 MB DOC)

Text S1 Details of clinical evaluation, including medical history and laboratory investigations, used for the subject selection.
(0.04 MB DOC)
