# Supplementary material for: Skeletal Site-Related Variation in Human Trabecular Bone Transcriptome and Signaling
Source: PLoS One. 2010 May 18;5(5):e10692. doi: 10.1371/journal.pone.0010692 (PMC2872667; doi:10.1371/journal.pone.0010692)
Supplement: Table S1 — Anatomical-site related comparison of overall molecular homogeneity between different skeletal sites. (0.01 MB .DOCX) [file pone.0010692.s001.docx]

**Table S1.** Anatomical-site related comparison overall molecular homogeneity between different skeletal sites.

**Table S2.** Examination of Gene Ontology (GO) terms show a significant over representation for a number of GO terms including biological and cell adhesion, extracellular matrix formation and skeletal development ((p<0.005).

**Table S3.** Top 30 Signalling Pathways identified by comparing differential transcript expression in the lumbar spine versus iliac crest (4244 Gene Transcripts with FC ≥ 2, p-value ≤ 0.05).

**Table S4.** TNF receptor signalling pathway gene transcripts identification based on differential expression in lamina lumbar spine and iliac crest; the analysis was carried out using Pathway Architect Software and the pathway generated is shown in Fig 2.

**Table S5.** BMP signalling pathway gene transcripts identification based on the differential expression in iliac crest and in the lamina of lumbar spine; the analysis was carried out using Pathway Architect Software and the pathway generated is shown in Fig 3.

**Table S6.** Proteoglycan Syndecan signalling events identification based on analysis of differential expression in the lamina of lumbar spine and iliac crest; the analysis was carried out using Pathway Architect Software and the pathway generated is shown in Fig 4.

**Table S7.** Probesets with a 5-fold or more change in expression were analysed with Ingenuity Pathway Analysis (IPA), a list of 268 eligible entities for IPA analysis. The most significant biological function classification was in skeletal and muscular development and function.

Table S8. Anthropometric indices age and bone density of individual subjects.

.
