# Supplementary material for: Skeletal Site-Related Variation in Human Trabecular Bone Transcriptome and Signaling
Source: PLoS One. 2010 May 18;5(5):e10692. doi: 10.1371/journal.pone.0010692 (PMC2872667; doi:10.1371/journal.pone.0010692)
Supplement: Table S3 — Top 30 signalling pathways identified by comparing differential transcript expression in the lumbar spine versus iliac crest (4244 gene transcripts with FC ≥2, p-value ≤0.05). (0.06 MB DOC) [file pone.0010692.s003.doc]

| **Object Identifier** | **Common objects** | **Size** | **Name** |
| --- | --- | --- | --- |
| 2108038 | 43 | 793 | TNF receptor signaling pathway |
| 2108042 | 42 | 554 | BMP Signaling |
| 2108035 | 34 | 515 | Proteogylcan syndecan-mediated signaling events |
| 2108037 | 34 | 459 | p38 MAPK signaling pathway |
| 2108036 | 32 | 555 | IL1-mediated signaling events |
| 2108041 | 31 | 797 | Class I PI3K signaling events |
| 2108020 | 31 | 390 | Regulation of p38-alpha and p38-beta |
| 2108021 | 25 | 448 | Endothelins |
| 2120434 | 18 | 215 | Hemostasis |
| 2108010 | 18 | 160 | Hypoxic and oxygen homeostasis regulation of HIF-1-alpha |
| 2107959 | 17 | 135 | HIF-1-alpha transcription factor network |
| 2107996 | 17 | 239 | Role of Calcineurin-dependent NFAT signaling in lymphocytes |
| 1625623 | 16 | 140 | Breast Cancer |
| 2108034 | 16 | 384 | Sphingosine 1-phosphate (S1P) pathway |
| 2108016 | 16 | 205 | Syndecan-2-mediated signaling events |
| 2120429 | 15 | 260 | Signaling in Immune system |
| 2107971 | 14 | 107 | Signaling mediated by p38-alpha and p38-beta |
| 2108000 | 14 | 161 | IL4-mediated signaling events |
| 2107956 | 14 | 89 | FOXM1 transcription factor network |
| 2108033 | 14 | 533 | p75(NTR)-mediated signaling |
| 2108025 | 13 | 389 | TCR signaling in naïve CD4+ T cells |
| 2120436 | 13 | 162 | Cell Cycle, Mitotic |
| 2108039 | 13 | 242 | Signaling by Aurora kinases |
| 2108013 | 13 | 178 | Signaling events mediated by the Hedgehog family |
| 2108040 | 13 | 299 | IL2-mediated signaling events |
| 2120411 | 13 | 130 | Apoptosis_1 |
| 2108008 | 12 | 129 | Syndecan-4-mediated signaling events |
| 2108017 | 12 | 305 | Neurotrophic factor-mediated Trk receptor signaling |
| 2108003 | 11 | 284 | Signaling events medaited by HDAC Class I |
| 2108015 | 11 | 183 | Integrins in angiogenesis |
