# Supplementary material for: Skeletal Site-Related Variation in Human Trabecular Bone Transcriptome and Signaling
Source: PLoS One. 2010 May 18;5(5):e10692. doi: 10.1371/journal.pone.0010692 (PMC2872667; doi:10.1371/journal.pone.0010692)
Supplement: Table S5 — BMP signalling pathway gene transcripts identification based on the differential expression in iliac crest and in the lamina of lumbar spine; the analysis was carried out using Pathway Architect Software and the pathway generated is shown in Fig. 3. (0.06 MB DOC) [file pone.0010692.s005.doc]

| **Gene Symbol** | **p-value**  **([LS] Vs [ILIAC])** | **FC Absolute**  **([LS] Vs [ILIAC])** |
| --- | --- | --- |
| ETV1 | 6.77E-11 | 22.53 |
| EGR1 | 4.10E-05 | 12.63 |
| GREM1 | 4.92E-04 | 6.93 |
| RCAN2 | 2.34E-05 | 6.36 |
| RGMA | 0.003 | 5.94 |
| JUN | 3.56E-05 | 5.21 |
| FOS | 0.022 | 4.89 |
| SUSD2 | 4.54E-04 | 4.85 |
| CHRD | 4.51E-04 | 4.25 |
| NOG | 3.49E-05 | 4.12 |
| PTGS2 | 0.015 | 3.96 |
| EGR3 | 7.28E-05 | 3.57 |
| 232454_at | 5.65E-05 | 3.55 |
| 238394_at | 0.040 | 3.51 |
| PTPRK | 4.76E-05 | 3.45 |
| MAF | 0.040 | 3.39 |
| BCL2 | 0.018 | 3.18 |
| BAMBI | 1.50E-04 | 3.14 |
| HSPB1 | 0.004 | 2.9 |
| EGR2 | 0.025 | 2.66 |
| 238623_at | 0.004 | 2.61 |
| 1570628_at | 0.036 | 2.39 |
| MAP2K6 | 0.012 | 2.26 |
| CCND1 | 0.004 | 2.19 |
| ESR1 | 0.001 | 2.17 |
| DUSP1 | 5.66E-04 | 2.17 |
| MITF | 0.001 | 2.12 |
| MEF2C | 0.016 | 2.09 |
| DUSP16 | 0.021 | 2.07 |
| MAP3K1 | 0.006 | -3.95 |
| MAPKAPK5 | 0.018 | -3.53 |
| NOS2A | 0.023 | -2.54 |
| CREB1 | 0.024 | -2.41 |
| BCL2L1 | 0.022 | -2.41 |
| MAPK14 | 0.017 | -2.32 |
| IKZF1 | 0.005 | -2.15 |
| LOC650083 /// YWHAZ | 0.023 | -2.1 |
| EIF4E | 0.002 | -2.09 |
| IL8 | 0.010 | -2.09 |
| MAPK1 | 0.006 | -2.06 |
| CSNK1A1 | 0.001 | -2.03 |
| MAP3K7IP2 | 0.014 | -2.03 |
