# Supplementary material for: Skeletal Site-Related Variation in Human Trabecular Bone Transcriptome and Signaling
Source: PLoS One. 2010 May 18;5(5):e10692. doi: 10.1371/journal.pone.0010692 (PMC2872667; doi:10.1371/journal.pone.0010692)
Supplement: Table S6 — Proteoglycan Syndecan signalling events identification based on analysis of differential expression in the lamina of lumbar spine and iliac crest; the analysis was carried out using Pathway Architect Software and the pathway generated is shown in Fig. 4. (0.06 MB DOC) [file pone.0010692.s006.doc]

| **Gene Symbol** | **p-value**  **([LS] Vs [ILIAC])** | **FC Absolute**  **([LS] Vs [ILIAC])** |
| --- | --- | --- |
| PTN | 5.14E-08 | 14.85 |
| FN1 | 1.54E-07 | 10.45 |
| FGFR2 | 4.22E-04 | 9.12 |
| 1561657_at | 2.80E-04 | 8.42 |
| LAMA3 | 2.49E-04 | 6.78 |
| ADAM12 | 9.96E-04 | 5.56 |
| CDH2 | 5.74E-04 | 4.3 |
| MET | 4.81E-05 | 4.19 |
| 1565633_at | 0.014 | 4.02 |
| FZD7 | 8.29E-06 | 3.85 |
| BGLAP | 2.51E-04 | 3.78 |
| FGF2 | 0.005 | 3.77 |
| CAV2 | 9.25E-05 | 3.71 |
| EGFR | 7.46E-07 | 3.6 |
| MMP2 | 5.82E-05 | 3.58 |
| SPP1 | 0.004 | 2.97 |
| MUC20 | 0.032 | 2.9 |
| RUNX2 | 0.012 | 2.74 |
| WNT5A | 0.009 | 2.66 |
| SDC4 | 8.70E-04 | 2.57 |
| 222080_s_at | 0.036 | 2.48 |
| SDC2 | 0.008 | 2.46 |
| TNC | 0.006 | 2.33 |
| DOCK1 | 7.26E-04 | 2.26 |
| FGFR1 | 0.012 | 2.17 |
| GIPC1 | 0.002 | 2.03 |
| PTPRJ | 0.017 | -3.79 |
| VIL2 | 0.018 | -3.27 |
| EPB41 | 0.021 | -3.06 |
| CXCR4 | 0.002 | -2.56 |
| IL8 | 0.010 | -2.09 |
| MAPK1 | 0.006 | -2.06 |
| CSNK1A1 | 0.001 | -2.03 |
| BSG | 0.029 | -2.03 |
