# Supplementary material for: Skeletal Site-Related Variation in Human Trabecular Bone Transcriptome and Signaling
Source: PLoS One. 2010 May 18;5(5):e10692. doi: 10.1371/journal.pone.0010692 (PMC2872667; doi:10.1371/journal.pone.0010692)
Supplement: Table S8 — Anthropometric indices age and bone density of individual subjects. (0.05 MB DOC) [file pone.0010692.s008.doc]

| **Subject**  **ID** | **Age**  **(yr)** | **Weight**  **(kg)** | **Height**  **(cm)** | **BMD**  **Lumbar spine**  **(gm/cm^2^)** | **BMD**  **total femoral**  **(gm/cm^2^)** | **BMD femoral neck**  **(gm/cm^2^)** |
| --- | --- | --- | --- | --- | --- | --- |
| OST1 | 58 | 85.0 | 184 | 0.985 | 0.983 | 0.801 |
| OST2 | 35 | 82.5 | 182 | 1.070 | 1.029 | 0.867 |
| OST3 | 54 | 98.2 | 184 | 0.947 | 1.014 | 0.862 |
| OST4 | 41 | 101.1 | 178 | 0.886 | 1.103 | 0.930 |
| OST10 | 56 | 88.8 | 180 | 1.136 | 1.204 | 0.972 |
| OST11 | 43 | 94.3 | 165 | 0.972 | 0.882 | 0.810 |
| OST12 | 69 | 104.1 | 180 | 0.926 | 0.946 | 0.763 |
| OST13 | 56 | 95.6 | 172 | 1.118 | 1.271 | 1.202 |
| OST14 | 66 | 108.2 | 178 | 1.094 | 0.916 | 0.693 |
| OST16 | 43 | 81.5 | 167 | 0.760 | 0.820 | 0.620 |
| OST17 | 66 | 84.0 | 179 | 1.350 | 0.967 | 0.821 |
| OST18 | 51 | 91.5 | 175 | 1.198 | 1.315 | 1.067 |
| OST19 | 62 | 97.3 | 169 | 1.387 | 1.160 | 0.907 |
