# Supplementary material for: Skeletal Site-Related Variation in Human Trabecular Bone Transcriptome and Signaling
Source: PLoS One. 2010 May 18;5(5):e10692. doi: 10.1371/journal.pone.0010692 (PMC2872667; doi:10.1371/journal.pone.0010692)
Supplement: Text S1 — Details of clinical evaluation, including medical history and laboratory investigations, used for the subject selection. (0.04 MB DOC) [file pone.0010692.s009.doc]

**Supporting Information**

**Subject evaluation and exclusion criteria**

*Subjects*

The research participations were Caucasians men from Tyne and Wear County, U.K., and undergoing lumber spinal orthopedic surgery, without any evidence of recent or past history of osteoporosis related fractures. All the surgical procedures and biopsies were carried out by one of the co-authors (Mr. Paul Sanderson, Orhopaedic Surgeon, Newcastle General Hospital, Newcastle upon Tyne, U.K.).

In additional to routine laboratory investigation, other possible secondary causes of osteoporosis were excluded by detail medical history and examination. The following questionnaire was used:

*Family History*

Family history of osteoporosis and osteoporosis-related fractures without trauma were excluded.

*Social History*

The questions related to alcohol intake, smoking, excessive caffeine intake, lack of exposure to sunlight, malnutrition, past history of exposure to heavy metals (such as workers in shipyards), particularly low body weight and frailty.

*Medical History*

The history specifically excluded medical history of anorexia, weight loss, height loss, GI disorders, neoplastic disease, prolonged immobilization or poor physical activity, hypercalciuria, endocrine diseases, connective tissue disorders, renal disorders, past history of fracture as an adult.

*Medication history*

The subjects on chronic treatment with steroids, excessive thyroid hormone, anticoagulant, anticonvulsant, lithium, immunosuppressive drugs were excluded.
